# Fortified electrospun collagen utilizing biocompatible Poly Glycerol Sebacate prepolymer (PGSp) and zink oxide nanoparticles (ZnO NPs) for diabetics wound healing: Physical, biological and animal studies

**DOI:** 10.1016/j.reth.2024.05.009

**Published:** 2024-05-29

**Authors:** Ghazaleh Larijani, Kazem Parivar, Nasim Hayati Roodbari, Parichehr Yaghmaei, Naser Amini

**Affiliations:** a Department of Biology, Science and Research Branch, Islamic Azad University, Tehran, Iran; b Cellular and Molecular Research Center, Iran University of Medical Sciences, Tehran, Iran

**Keywords:** Poly Glycerol Sebacate prepolymer (PGSp), Zink oxide nanoparticles (ZnO NPs), Collagen, Diabetics wound, Electrospun, Nanofiber

## Abstract

Collagen, a naturally occurring fibrous protein, is a potential resource of biological materials for tissue engineering and regenerative medicine because it is structurally biocompatible, has low immunogenicity, is biodegradable, and is biomimetic. Numerous studies have documented in the literature how Collagen nanofibers exhibit limited cell adhesion, poor viscosity, and no interior fibril structure. The biomedical industry is using Poly Glycerol Sebacate prepolymer(PGSp), a biodegradable and biocompatible polyester with high adhesion and very viscous appearance, more often. Here, unique electrospun Collagen/PGSp/ZnO/NPs blend nanofibers for skin tissue application were developed and described with varied PGSp percent. Additionally, when ternary blends of Collagen, PGSp, and Zink Oxide Nanoparticles (ZnO NPs) are used, the antibacterial properties of the scaffolds are improved. The bead-free electrospun nanofibers were produced by raising the PGSp concentration to 30%w/w. SEM, EDS, tensile, MTT, FTIR, SDS-page, swelling test, contact-angle, antimicrobial, biodegradation, XRD, and cell attachment procedures were used to characterize the crosslinked nanofibers. The ternary blend nanofibers with a weight ratio of Collagen/PGSp 30%/ZnONPs 1% had higher stress/strain strength (0.25 mm/mm), porosity (563), cell survival, and degradation time. Moreover, after applying for wound healing in diabetic rats, Collagen/PGSp 30%/could be show improving wound healing significantly compared to other groups.

## Graphical abstract

**Figure:**
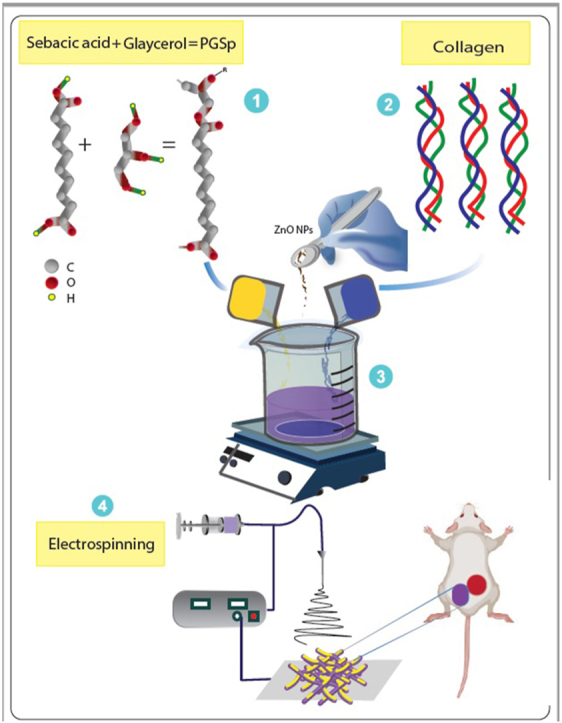

## 1 Introduction

Diabetic wound is one of the complications of diabetes which not only causes physical trauma but also resulted in social and economic burden for patients. Today, dressings and novel skin substitutes are used to treat these wounds. The principal barrier between the body and the environment skin is a multilayered tissue that is constantly exposed to the environment, making it extremely vulnerable to lesions of all kinds [1]. To facilitate cell growth and transfer nutrients, oxygen, metabolites, and growth factors, skin substitutes must have 3-D macro-porous structures with high porosity and pore interconnectivity [2]. Since Nanoscale fibers are formed with assembled micro- or nano-mesh structure that mimics the natural extracellular matrix, they have become an ideal structure for the preparation of wound scaffold, in contrast to traditional polymeric wound dressings like membranes, composites, sponges, or hydrogels (for Extra cellular Matrix (ECM) regeneration) [3,4]. Highly permeable scaffolds with customizable fiber diameter, scaffold thickness, biodegradability, and biocompatibility can be made using electrospun nanofibrous scaffolds [5].

The biomaterials currently used as matrices for wound repair can be classified into natural and synthetic types according to their origin. An ideal source of biological materials for tissue engineering and regenerative medicine is Collagen, a naturally occurring fibrous protein that is biocompatible structurally, has reduced immunogenicity, is biodegradable, and is biomimetic [6]. Collagen type I compensates between 70 and 90% of the Collagen in the body and is found in a variety of tissues as elongated fibers [7]. By combining excellent biological mimicry of the biological material with the fibrous shape of the protein, Collagen electrospun scaffolds effectively mimic the structure of the ECM of natural tissues [8,9]. Numerous studies have investigated electrospinning Collagen either alone [10,11] or in combination with other materials such as chitosan [8,12,13], PCL [14,15], elastin [16,17], nanohydroxyapatite [18], silk fibroin [19], polyurethane [20,21], etc. There are conflicting reports in the literature about low cell adhesion [22], low viscosity [16], and lack of internal fibril structure on Collagen nanofibers [23], which are all physical and biological features of electrospun Collagen fibers that are still being contested. The critical question is what can be added to Collagen to enhance its physicochemical qualities during the electrospinning process.

Poly Glycerol Sebacate prepolymer(PGSp) is a durable, biodegradable, and biocompatible elastomer made by polycondensing glycerol and sebacic acid [24]. PGSp mechanical characteristics, hydrophilicity, and degradation behavior can be modified by varying the monomer molar ratio, reaction temperature, and reaction time [25,26]. It is a thermoset elastomer with great elasticity and flexibility and a minimal inflammatory response that has a wide range of physicochemical, mechanical, and morphological qualities for a number of applications [27].

PGSp metabolic byproducts are harmless and can be eliminated by the body. Because of these beneficial properties, PGSp has emerged as an excellent choice for soft tissue engineering applications such as vascular grafting [28], nerve guidance [29], cartilage tissue repair [30,31], retinal transplantation [32], bone tissue engineering [33,34] and wound healing [35]. The biomedical industry is using PGSp, a biodegradable and biocompatible polyester with light yellow and very viscous appearance, more often. As a result, in this investigation, we utilized various percentages of PGSp to improve the physicochemical properties of Collagen prior to electrospinning.

Zink Oxide Nanoparticles (ZnO NPs) have been added to various wound substances to provide antibacterial characteristics [36]. The US Food and Drug Administration has classified ZnO NPs as “generally recognized as safe” (21 CFR182.8991). Metal nanoparticles such as ZnO NPs have different chemical, mechanical, electrical, structural and morphological properties than zinc oxide. These modified characteristics make interact better with cellular biomolecules and the physical transfer of ZNO NPs into cellular structures is also easier [37]. Previous research has shown that zinc oxide nanoparticles have potent antibacterial characteristics, are biocompatible, self-cleaning, compatible with the skin, and sunscreens, and can be used for a variety of biomedical specialty purposes [38]. Collagen, ZnO NPs, and various PGSp concentrations were combined in this investigation, and the mixture was electrospun. These composite scaffolds' composition, microstructure, porosity, water contact angle, tensile strength, swelling, degradation, anti-bacterial properties, toxicity, and cell adhesion were all examined. The goal of this work was to create an electrospun Collagen scaffold for use in treating wounds that had improved physical and biological characteristics. Moreover, In vivo assessment was done for better understanding of the effects of the scaffolds on wound healing in the diabetic rats.

## 2 Methods and material

### 2.1 Material

Sigma Aldrich provided the following chemicals: pepsin (CAS #: 9001-75-6), ethylenediaminetetraacetic acid (EDTA) (CAS #: 60-00-4), and disodium hydrogen phosphate (Na2HPO4) (CAS #: 7558-79-4). (Sigma Aldrich Chemical Co, Steinheim, Germany). Calcium chloride (CAS #: 10043-52-4), glutaraldehyde 25% (CAS #: 111-30-8), sodium chloride (MW = 58.44 g/mol), acetic acid 100% (CAS #: 64-19-7), ethanol 96%, dimethyl sulfoxide (DMSO, CAS #: 67-68- 5), and sebacic acid (CAS #: 111-20-6) were all acquired from Merck (Darmstadt, Germany). The following items were purchased from Gibco in the USA: DMEMF12(Dulbecco's Modified Eagle Medium F12, CAS#: 31330), Penicillin-Streptomycin (10,000 U/ml, CAS#: 3810-74-0), PBS (phosphate buffer saline, CAS #: 7778-77-0), and FBS (fetal bovine serum, CAS#: 1943609-65-1). The following items were purchased from Sigma-Aldrich in the USA: (3–4, 5-dimethylthiazol-2-yl-2, 5- diphenyltetrazolium bromide) (MTT), zinc nitrate (CAS# 10196-18-6), and glycerol (CAS#: 56-81-5).

### 2.2 Isolation of collagen

The bovine Achilles tendon was used to obtain Collagen. Each Achilles tendon was stored in ethanol up to processing, and then it was sliced into small parts (1 cm) and cleaned with deionized water for 48 h (1 g tendon/10 ml H2O), which was changed five times each day. All processes were carried out at 4 °C with constant stirring. The washing was then carried out for 48 h with 0.05 M TRIS and 1 M NaCl (pH 7.5), with this solution being changed twice daily. The tendon was then homogenized in 0.83 M acetic acid and dissolved there for 36 h before being introduced to pepsin (0.24%) and hydrolyzed there for two days. The hydrolysis was subsequently stopped by raising the pH to 7.5 with 1.0 M NaOH. After 12 h, the homogenate was centrifuged for 30 min at 10,000 g. The supernatant was saliently precipitated by adding up to 3 M of NaCl. After being precipitated for 24 h, the solution was centrifuged at 10,000 g for 45 min, and the pellet was then re-suspended in 0.80 M acetic acid and dialyzed in membrane dialysis against 0.5 M acetic acid for one week with a daily replacement of the dialysis solution. The dialyzed Collagen was then lyophilized and kept at 20 °C until needed [39].

### 2.3 Characterizations of collagen

#### 2.3.1 Energy dispersive x-ray spectroscopy (EDS)

Energy dispersive spectroscopy (EDS) is a microanalytical technique used to identify and quantify elements in samples. Using an Oxford Inca Energy 200 system connected to a JOEL JSM 6360LV scanning electron microscope, nitrogen mapping by Energy Dispersive X-ray (EDS) microanalysis was performed at 20 kV for 20 min. To observe the distribution of this element, which is present in Collagen, nitrogen EDS mapping was done [40].

#### 2.3.2 Fourier transform infrared (FTIR) spectroscopy

A Bruker FT-IR spectrometer (Billerica, Massachusetts, United States) was used to perform FT-IR spectroscopy in order to evaluate the molecular characteristics of several materials (Collagen, PGSp, and electrospun fibers). Powdered and compacted into clear potassium bromide disks, the electrospun fiber samples were scanned in the transmittance mode from 4000 to 500 cm^−1^ [41].

#### 2.3.3 SDS-polyacrylamide gel electrophoresis (SDS-PAGE)

Collagens were processed to the discontinuous Tris-HCl/glycine buffer technique of Laemmli (1970), with 7.5% separating and 5% stocking gel. At 95 °C (1.5 mg/1 ml), Collagen samples were first dissolved in a samples buffer. The derbies then were taken out following a 5-min, 4000 rpm centrifugation using a mini centrifuge. Samples were then placed onto a polyacrylamide gel and treated to electrophoresis using a Mini Protein II instrument coupled with high MW markers (Bio-Rad Laboratories, Inc., Richmond, CA, USA). The gel was first stained with 0.05% Coomassie blue R-250 in 15% (v/v) methanol and 5% (v/v) acetic acid, followed by destained with 30% (v/v) methanol and 10% (v/v) acetic acid after electrophoresis [42].

### 2.4 Preparation PGSp

According to the Wang et al. published technique, and as shown in Fig. 2A the PGSp was synthesized. In a nutshell, 0.1 M sebacic acid and 0.1 M polyol glycerol were combined, then heated at 120 C under an inert nitrogen environment for 24 h to produce PGSp. The reaction then proceeded for 48 h at the same temperature under gradually increasing vacuum pressure (almost 50 mTorr) until PGSp was produced [43,44].

### 2.5 X-ray diffractometer (XRD)

The PGSp were examined using an X-ray diffractometer (XRD, Bruker AXS, Karlsruhe, Germany) with a 20° diffraction angle from 50 to 400° and a 10 Hz scanning rate at room temperature. The physical state of PGSp was examined [45].

### 2.6 Prepation zink nanao oxide

The basic ingredients zinc nitrate and urea, both analytical-grade products, were used to create nano-zinc oxide nanocrystals. The precipitant was employed as urea, and the molar ratio of urea to zinc nitrate was 1:2. The following steps were taken during the preparation process to produce nanometer-sized zinc oxide powders: first, the solutions were heated to 95° Celsius for 4 h while under saturated vapor pressure. Next, the precipitates were dried for 24 h at room temperature (25° Celsius). Finally, 500° Celsius was used for thermal treatment [46].

### 2.7 Preparation of electrospinning solution

A nanofibrous scaffold made of a PGSp/Collagen mixture was created. Separately, Collagen and PGSp were dissolved in acetic acid 0.5 M at room temperature (with the thermostat set at 25 C) while being stirred continuously for 1 h. Before electrospinning, the mixture was gently stirred for 1 h at room temperature while being combined with the Collagen/PGSp ratios of 90:10, 70:30, and 50:50. As an antibacterial agent, we added 1% ZnO NPs to all solutions. PH of final solution was 7.2.

### 2.8 Electrospinning process

The syringe pump was equipped with a 5 mL syringe with a connection tube and a 21-gauge blunt-ended needle. 24 kV, 0.5 mL/h21, and 20 cm were used as the voltage, feed rate, and collector distance, respectively. After 8 h, the electrospinning process was halted. The electrospun sheets were subjected to 0.4 bar oxygen (O2) pressure for 5 min in a 30 W microwave plasma machine chamber to plasma treat for surface modification (Diener Electronics, Germany). The scaffolds were kept at 220C until it was ready for cell culture, at which point they were cleaned with PBS and 70% ethanol and exposed to UV light for 20 min.

### 2.9 Cross-linking of nanofibers

The PGSp/Collagen nanofibrous membrane was crosslinked using the following technique: glutaraldehyde vapor, which involved adding 40 mL of 25% (v/v) glutaraldehyde to a container, putting the nanofibrous membrane on top, and sealing the container with a hollowed-out lid to allow vapor flow for 18 h. This procedure produced insoluble nanofibers inside a chamber with a humidity of 55%. Then scaffolds rinsed three times with distilled water [47].

### 2.10 Characterization of electrospun nanofibers

#### 2.10.1 Analysis of scanning electron microscope (SEM)

The fibers were subjected to SEM examination in order to investigate just at the shape and microscopic structure of the nanofibers as well as to calculate their diameter. The fibers were initially cut into 1 cm^2^ squares, placed on aluminum foil, and then ready for SEM investigation. Electrospun nanofibers were punched and placed the 24-well culture plates. Adipose-Derived Stem Cell (ADSC) were placed at 3 × 104 cells in each nanofiber and cultured for 2 days at 37 C in 5% CO2. We used a Czech-made TESCAN VEGA electron microscope for the ongoing project. The divided sample fibers were coated with 900 Å of gold prior to imaging. In SEM examination, electron beams are placed on the sample fibers' surfaces, and the emissions are then scanned while a picture of the samples' surfaces is displayed on a monitor. Seven different zooms were used to scan the fibers in order to better understand their shape [20].

#### 2.10.2 Swelling test

The weights of dried (Wdry) and wet mats (Wwet) were used to calculate the swelling capacity. Mat ribbons (1 cm 2 cm) were submerged in deionized water for 48 h at 37 °C in an incubator. The resulting nanofibers were weighed again after being stained with filter paper to remove extra surface water. The proportion of weight determined using the following formula is used to represent the scaffold's water uptake:

Swelling(%) = W2–W1/W1∗ 100

W1 = weight of the polymer (afore swelling) W2 = weight of the polymer (later swelling) [48].

#### 2.10.3 Water contact angle measurement

In order to examine the hydrophilicity of the electrospun PGSp/Collagen membranes and provide insight into the scaffold's wetting capacity, water contact angle measurements were conducted. Images were captured with a Canon EOS 700D camera and an EF-S 60 mm f/2.8 Macro USM zoom lens utilizing droplets with a volume of 3 L that were deposited onto surfaces. MB ruler 5.3 software was used to measure the contact angles [49].

#### 2.10.4 Mechanical performance

Using a universal tension tester, the mechanical characteristics of electrospun membranes were evaluated (Instron 5967 USA). The membranes were divided into rectangle samples of 50 mm by 20 mm, with a thickness control set at around 0.05 mm, and the thickness was measured using a micrometer caliper, with an average error of 0.01 mm. At a tensile rate of 10 mm/min, the uniaxial tensile test was conducted. Five samples were used to compute the average tensile strength and elongation at break [45].

#### 2.10.5 In vitro cytocompatibility of electrospun membranes

The cytotoxicity of scaffolds was evaluated using a direct MTT assay. Samples of the composite nanofibers were first sterilized by UV light for this purpose. The nanofiber scaffolds were subjected to an MTT test in a 96-well plate. The scaffolds constructed in the plate wells received 20 l of a fibroblast cell solution comprising 10,000 cells. After 30 min of incubation, 90 μl of culture media were added to the wells with the cells. 100 μl of culture media was once again supplied to the cells after 3 h to keep them from suspending and encourage their adhesion to the scaffolds. To assess the color diffusion into the media, certain wells were assigned to scaffolds without cells. Incubation of the cell culture plate took place for 1, 3, and 5 days. To distinguish between cells that adhere to scaffolds and those that remain on the bottom of the wells, the scaffolds were placed in empty wells on a plate. The culture medium in the wells was then drained, and 200 μl of a solution containing MTT color with a 0.5 mg/ml concentration was dissolved into sterilized culture media and applied to each well. For 4 h, the well plate was incubated. An insulin syringe was used to remove the medium from the top of the cells and the scaffolds after centrifuging the plates to adhere the precipitate to the bottom of the wells. Each well received 200 μl of DMSO, which was then mixed with a magnetic stirrer. The scaffolds were removed from the plates once the MTT color particles had thoroughly dissolved, and Elisa Reader evaluated the optical absorbance at 570 nm. Lastly, the number of viable cells was determined using the formula below:

Percentage of cell viability(%) = OD(s)-OD(blank)/OD(control)-OD(blank) (8).

#### 2.10.6 SEM and cell attachment

Cells were cultivated over a nanofibrous Collagen construction, and SEM was used to analyze their morphological characters. Punches (12 mm in diameter) containing electrospun Collagen nanofibers were put and dropped onto 24-well culture plates. Each electrospun Collagen nanofiber was coated with 2 × 10^4^ Adipocyte-Derived Stem Cells (ADSC), which were then cultivated for one or three days at 37 °C with 5% CO2. The experimental wells were rinsed twice with PBS to eliminate any light adhesions or loose cells. The remaining certain cells were then preserved in 2.5% glutaraldehyde for 5 min. Finally, the fixative was eliminated. Collagen nanofibers electrospun were formerly dehydrated in a classified sequence of ethanol solutions after being rinsed in PBS. The samples were sputtered using an SEM coating system after critical point drying. Using a sputter coater, gold was applied to nanofiber mats. Using ImageJ analysis software, the average fiber diameter was calculated [50].

#### 2.10.7 Antimicrobial activity

*Staphylococcus aureus* and *E. coli* bacterium strains were tested as Gram-positive and Gram-negative, respectively, to examine the antibacterial activity of the mats with equal weight ratios of ZnO NPs (1% wt) against two harmful bacteria. The 2108 CFU.mL-1 bacterial suspensions were grown on Mueller Hinton Agar plates using the agar disc diffusion method. Then, agar plates with square mat disks on them were positioned there for an overnight incubation period at 37 °C. As a consequence, the inhibition zones surrounding the mats containing ZnO NPs that indicate antibacterial activity were examined three times using a caliper [48].

#### 2.10.8 Degradation rate

Dry scaffolds are used to quantify deterioration as a percentage of weight loss. Three sets of scaffolds were combined in PBS and incubated in cell culture under different time constraints (0, 7, 14, and 21 days). Three groups of scaffolds were weighed before the test began, and each scaffold was weighed once a week after being taken out of the medium. Prior to measurement, the scaffolds are vacuum-dried. A percentage of the starting weight is calculated and used to represent weight loss. W1 and W2 are the weights of the specimens before and after the deterioration, and the following formula calculates the percent weight loss of the samples from the weights acquired before and after the destruction [51].

Weight loss % = (W1–W2)/W1 × 100

### 2.11 In vivo assessment

In this study 32 Albino Wistar rats weighing 200–250 g were used. Animals were housed in polycarbonate cages (temperature: 25 ± 2 °C, 12-h light/12-h dark cycle and a humidity of 60–70%). The rats were given standard pellet chow and tap water [15,16]. The protocol was under an IACUC approved research protocol at Islamic Azad University (Ethical code: IR.IAU.SRB.ERC.1400.168) and were in accordance with the policy on Humane Care and Use of Laboratory Animals and Guide for the Care and Use of Laboratory Animals.

In the first step for inducing diabetes, an intraperitoneal injection of Streptozotocin (cat number:18883-66-4) with the dose of 50 mg/kg prepared in saline was done for each rats. Three days after the injection, hyperglycemia was confirmed by measuring the blood glucose of rats using glucometer (blood was collected from the end of their tails). Animals showing fasting blood sugar levels more than 200 mg/dL were considered to be diabetic.

The diabetic rats were anesthetized by ketamine Sigma Aldrich (St Louis, MO, USA) (91 mg/kg) and xylazine (Sigma Aldich, Steinheim, Germany) (9 mg/kg) combination intraperitoneally. The hair on their back (dorsum) was shaved and wound field was outlined with a marker before removing the skin. A circled field having 1.5 cm (15 mm) diameter was drawn on their dorsum by marker. By using the appropriate surgical equipment, a deep skin wound was created via removing a circle of skin with 15 mm diameter on their dorsum while removing the subcutaneous connective tissue. Each rats had two holes drilled into its back. Wounds were studied in five groups (each group: n = 6). The first groups were treated with Collagen + ZnO NPs nanofiber. The second, third and fourth groups were treated with Collagen/PGSp10%/ZnONPs, Collagen/PGSp 30%/ZnONPs, and Collagen/PGSp 50%/ZnONPs nanofibers, respectively. For the control group, the wound was covered with gaze.

Before treatment, all wounds were cleaned daily with sterile saline. Additionally, all rates were kept in standard laboratory animal cages to prevent possible infection or scratching. Moreover the body weight of the rats was measured (days 0, 7, and 14). The wound area images were captured after 0, 7, 14 days by the DSCW320 Sony® digital camera (Sony, Tokyo, Japan). The rats were sacrificed by CO2 inhalation, and the wound area was harvested after 7 and 14 days and kept for analyzing the area of the skin grafts and the surrounding tissues with histopathologic techniques such as H&E and Masson staining. The pathological evaluation was done as well. The wound healing was evaluated by measurement of wound surface area using caliper Vernier and the ImageJ software (Version 1.8.0.) after treatment initiation in appropriate time points (days 7 and 14). Moreover, the healing ratio was calculated by this equation: Healing ratio (%) = 100 × (1 – wound area after treatment/initial wound area) [21,23].

### 2.12 Statistical analysis

Version 9 of the Prism statistical analysis program was used for the calculations. Results that had a p-value under 0.05 were considered significant. For each reported piece of data, average values and standard deviations are provided. One way ANOVA and Tukey HSD were used for statistical analysis.

## 3 Results and discussion

### 3.1 Characterization of extracted collagen

**Fig. 1:**
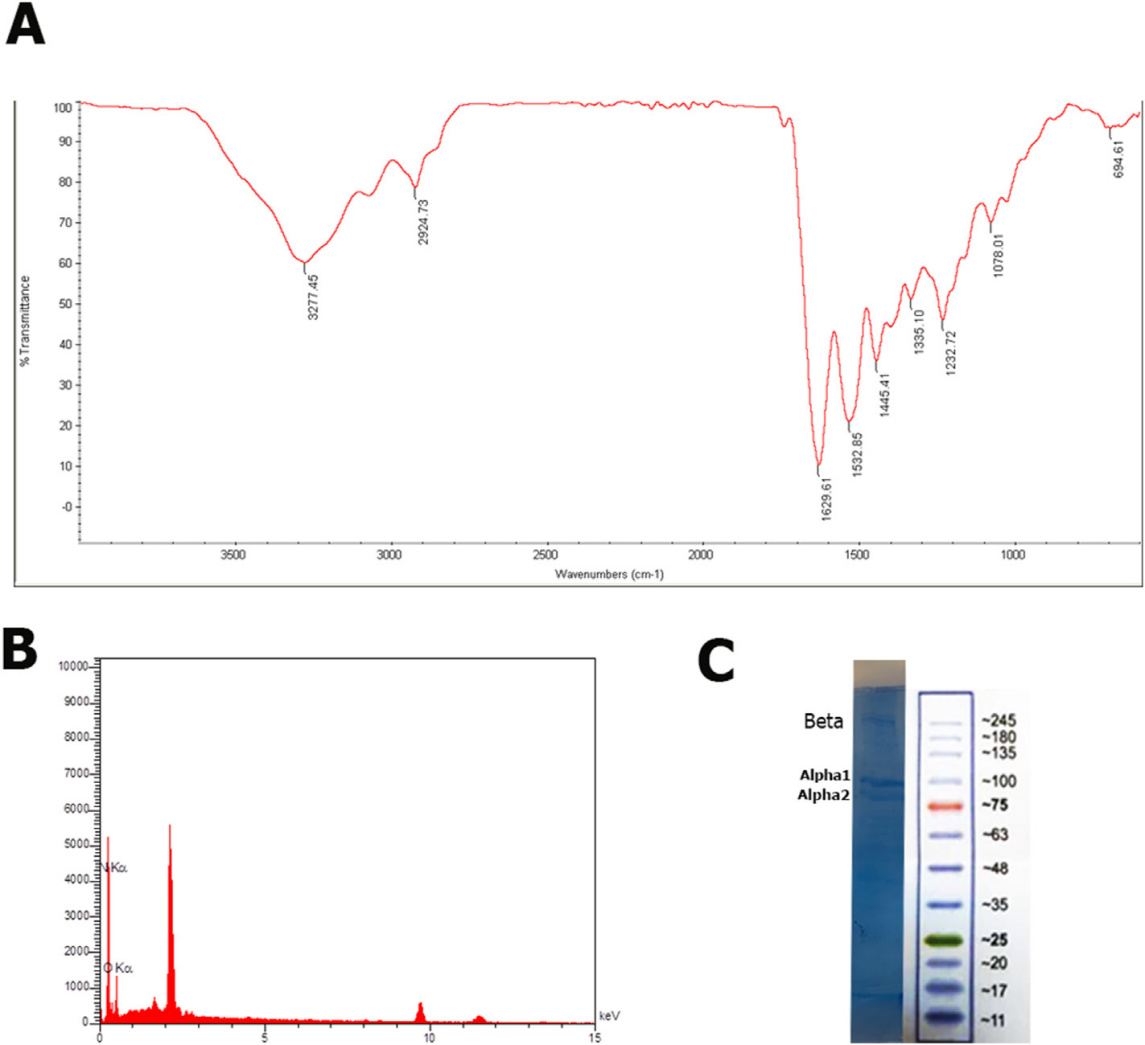
Collagen characterization: A, Fourier transform infrared (FTIR) spectra of Collagen extracted from Bovine tendon, B, Energy dispersive x-ray spectroscopy (EDS) diagram of the extracted Collagen, C, SDS-Polyacrylamide gel electrophoresis (SDS-PAGE) of the extracted Collagen.

Collagen's structures, particularly its functional groups and chemical bonds, are frequently studied using FTIR spectra. Fig. 1A displays FTIR spectra of Collagen from bovine tendons. The principal peaks in the Collagen spectra from bovine tendons, such as A, B, I, II, and III amides, matched those of type I Collagen [52]. Collagen's amide A has shown asymmetrical stretching of the NH atom, and hydrogen bonds in the around 3200–3440 cm^1^ range have been discovered. The Collagen's CH2 asymmetric stretch, which ranges from 2935 to 2915 cm^1^, is amenable to amine B absorption. The absorption zone was between 1600 and 1690 cm^1^, and the amide I C=O stretch was seen at 1681.02 cm^1^. For CN stretching and NH bending, there are amide II bands. The center of the amide II spectra, which occurred in the 1480–1575 cm^−1^ absorption range, was at 1553.71 cm^−1^. At 1450.52 cm^1^, the CH2 bend area of amide II was identified. The area between 1229 and 1301 cm^1^ was the location of the amide III NH bending of the NH linked to the CN group. The amide III in the Collagen samples was found using a wave number of 1234.48 cm^1^. The EDS analysis of the isolated Collagen is shown in Fig. 1b. Carbon (C = 50.17 0.32%), nitrogen (N = 21.07 0.52%), and oxygen (O = 27.19 2.92%) were the three principal components identified by elemental analysis using EDS in Collagen, which according to Fauzi et al. closely correlates to bovine tendon Collagen type 1 [53]. According to the findings, the Collagen from this study's extraction is extremely comparable to the Collagen from our earlier study's Collagen from the bovine tendon [9].

**Fig. 2:**
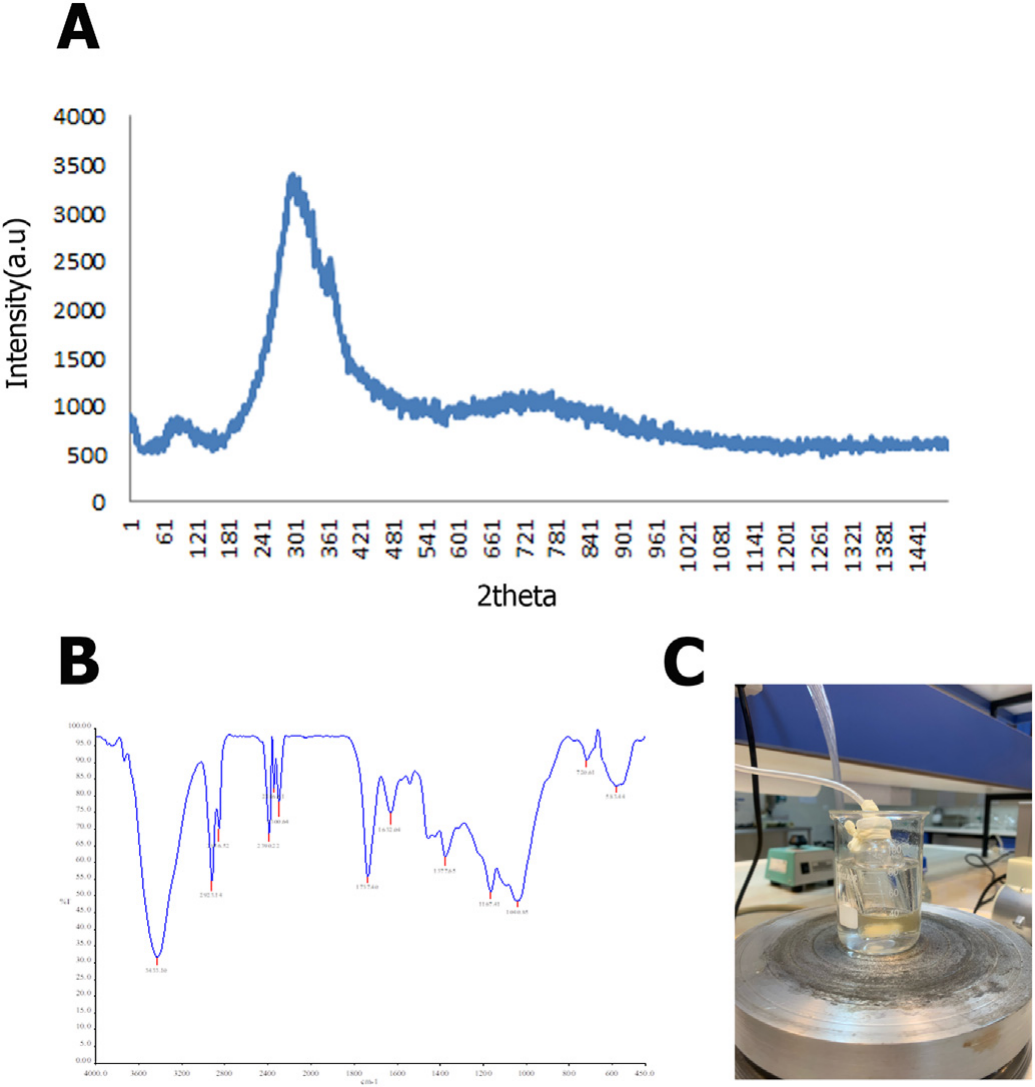
Poly Glycerol Sebacate prepolymer (PGSp) characterization: A, X-ray Diffractometer (XRD) of PGSp was synthesized in 7 h at 170 °C, B, Fourier transform infrared (FTIR) spectra of PGSp, C, PGSp was synthesized with 0.1 M sebacic acid and 0.1 M polyol glycerol combine.

Fig. 1C displays the SDS-PAGE arrangement of Collagen from the bovine tendon. In general, type I Collagen has a triple helix structure made up of two -chains (-chain and -chain) and one -chain [54]. Collagen's SDS-PAGE pattern revealed three distinct bands at 90 kDa (1-chain), 110 kDa (2-chain), and 240 KDa (-chain). According to Shi and Cuiping's findings, type 1 Collagen from cows has chains with diameters of 100, 130, and 215 kDa. The SDS-PAGE findings showed that the isolated Collagen was comparatively pure Collagen.

### 3.2 Characterization of PGSp

Fig. 2B displays the PGSp’ Fourier transform infrared (FTIR) spectra. PGSp was synthesized under nitrogen gas for 24 h at a steady temperature. OH, stretching is responsible for the peak at 936 cm1, whereas OH bending can be seen at 1410 cm^1^. Methylene (CH2) groups are responsible for the two peaks at 2854 and 2926 cm-^1^, while the strong peaks at 1181 and 1733 cm^−1^ are related to C=O and CO production, respectively [55]. The carbonyl stretching of the unreacted free sebacic acid is related to the sharp peak seen at 1691 cm^1^. To create a PGSp, Saudi et al. used a variety of times at a constant temperature of 170°; they recommended 7 h at this temperature [56]. In our investigation, the sharp peaks were achieved after 24 h of operation at a temperature of 120°. As a result, we employ it in the electrospinning procedure. Since the PGSp is completely amorphous, XRD might be used to detect how crystalline the mixed fibers are [57]. In Fig. 2A the fundamental patterns are displayed. The PGSp XRD pattern shows two peaks at 300 and 360. Given that the pre-polymer is widely recognized as an amorphous polymer, the signals are surprisingly sharp [20,42].

### 3.3 Nanofibers characterization

#### 3.3.1 Morphological analysis

**Fig. 3:**
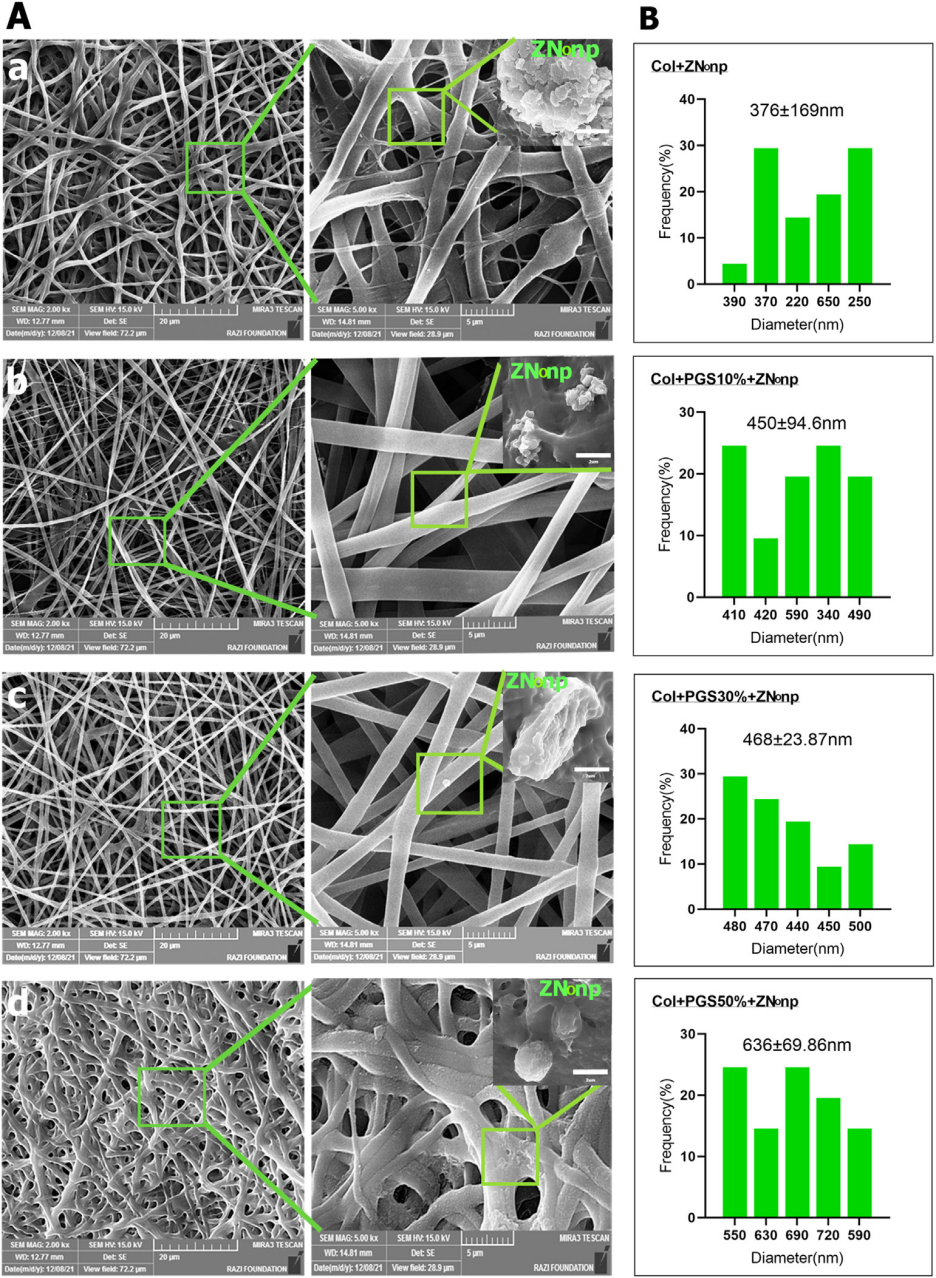
SEM images (A and fiber diameter distribution (B) of the nanofiber samples: (a) Col/ZnONPs (b) Collagen/PGSp10%/ZnONPs (c) Collagen/PGSp30%/ZnONPs (d) Collagen/PGSp50%/ZnONPs.

SEM photos showed the surface morphologies of electrospun Collagen mats developed at various PGSp concentrations. Table 1 demonstrates that while the scaffolds were of the same thickness and the fiber densities were similar, the Collagen/PGSp30%/ZnONPs mats had a higher percentage of pores. Fig. 3A and B showed that in Collagen/PGSp 30%/ZnONPs mats, porous fibers had good interconnected porous architectures and continuous structures with mean fiber diameters of 468 nm and porosities of 56%. Collagen/PGSp 50%/ZnONPs scaffolds with 50% PGSp had large fiber diameters (636 69 nm) and few pores (34% 4). Additionally, the scaffold holes were smaller in the Collagen/PGSp 10%/ZnONPs group because the Collagen fibers had a lower porosity (27%1) and a more uniform diameter (45094 nm) than the Collagen/PGSp 30%/ZnONPs mats did. The Collagen/ZnONPs group had a smaller fiber diameter (376169 nm) and porosity (20%2) than the other groups. Therefore, it may be inferred that scaffolds with smooth surface morphology and a higher percentage of porosity can be produced by adding additional PGSp to Collagen up to 30% more. Bead formation in electrospun nanofibers was seen in small numbers in all scaffolds. The formation of these beads happened due to the variability of the polymer solution jet and beaded fibers can be effective in biomedical scaffolds such as drug loading. The high viscosity of PGSp has been highlighted in earlier articles [2,26,56]. can be related to the aligned electrospun fibers and increased fiber fusion of the Collagen scaffold. PVA-PGS (50:50 and 40:60) fibers were demonstrated by Saudi et al. to enable the construction of scaffolds with homogeneous fiber diameters and no beads [56].

**Table 1:** Details on thickness, density, and porosity of Collagen/PGS electrospun materials at different ratios.

Collagen/PGS Blend	100/0	50/50	70/30	90/10	P-Value
Thickness(mm)	0.051 ± 0.003	0.053 ± 0.002	0.055 ± 0.004	0.053 ± 0.003	n.s
Apparent density of scaffold(g/cc)	0.41 ± 0.03	0.49 ± 0.02	0.59 ± 0.02	0.50 ± 0.04	0.08
Porosity (%)	20 ± 2	34 ± 40	56 ± 3	27 ± 1	0.02∗

#### 3.3.2 FTIR spectroscopy

**Fig. 4:**
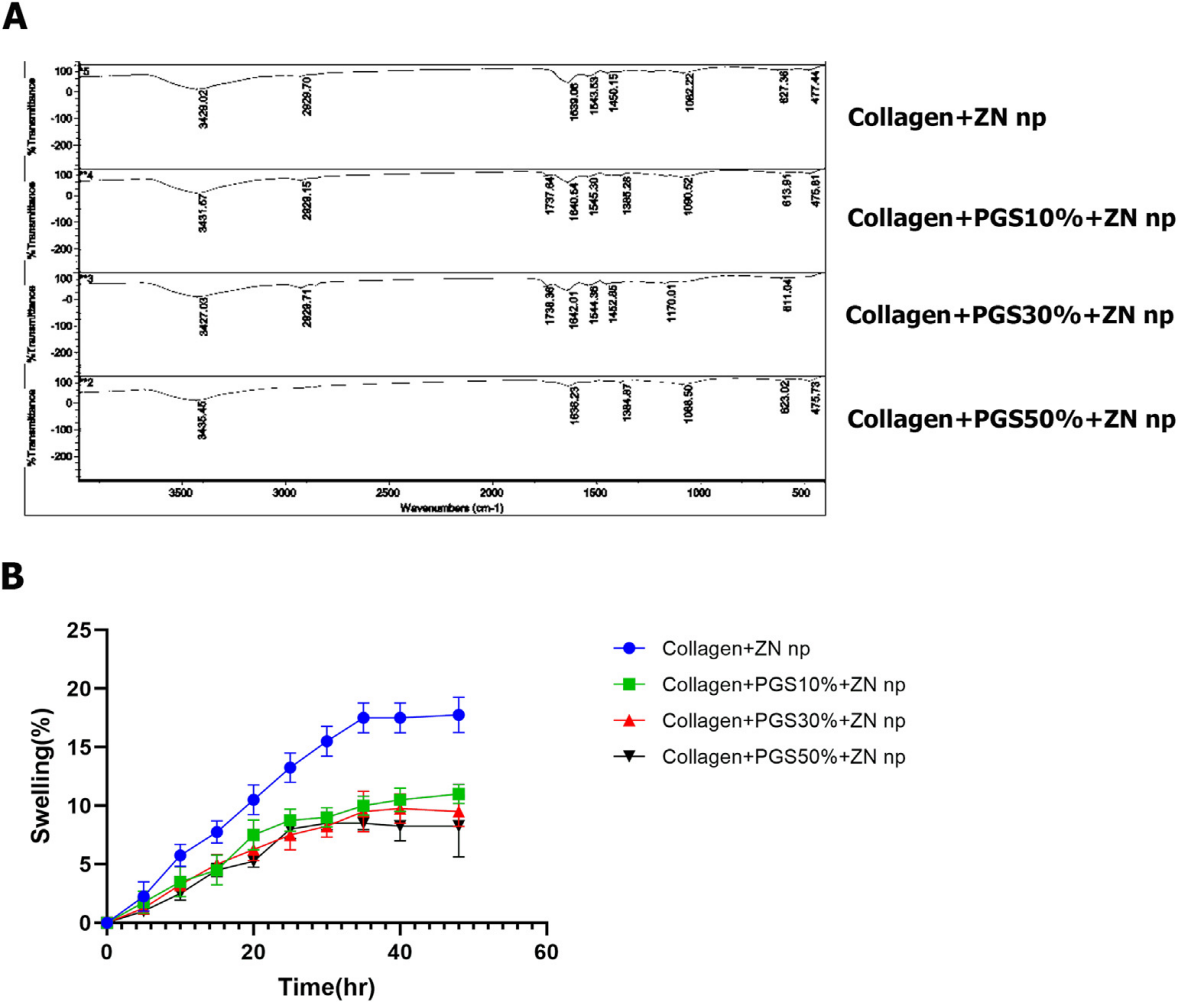
Nanofibers characterization: A, Fourier transform infrared (FTIR) spectra of nanofibers synthesized in different of PGSp percents, B, Swelling degree in PBS for Col/ZnONPs, Collagen/PGSp10%/ZnONPs, Collagen/PGSp30%/ZnONPs, and Collagen/PGSp50%/ZnONPs.

Collagen/ZnONPs, Collagen/PGSp 10%/ZnONPs, Collagen/PGSp 30%/ZnONPs, and Collagen/PGSp 50%/ZnONPs nanofibers' chemical structures were studied by FTIR, as shown in Fig. 4A. The N–H stretch amide peak at 3395 cm^−1^, the C–O stretch amide peak at 1647 cm^−1^, the N–H stretch amide peak at 1240 cm^−1^, and the C–O stretching vibration peak at 1081 cm^−1^ are all indicative of Collagen/ZnONPs nanofiber. Spectrums of Collagen, PGSp, and ZnONPs show peaks at 1650 cm^−1^ for C=O (amide I band), 1542 cm-1 for C–N (amide II), 1244 cm^−1^ for amide III, and 963 cm^−1^ for -CH2. These peaks are attributable to the presence of Collagen [27].

#### 3.3.3 Swelling test

Fig. 4B displays the swelling ratios of electrospun nanofibers. Compared to other scaffolds, Collagen/ZnONPs nanofiber showed a significant swelling ratio. As seen in Fig. 4A. For intervals of 10, 20, 30, and 40 h, respectively, Equation 1 produced swelling degrees of 4.4.04.5%, 7.14.3%, 8.22.71%, and 9.43.15% for the Collagen/PGSp electrospun samples. While Collagen is a highly hydrophilic polymer and makes up the majority of the nanofibers, PGSp is a hydrophobic polymer that makes up a smaller portion of the samples [58,59]. Additionally, adding PGSp to Collagen reduced its capacity to absorb water; however, doing so also lengthened the time it took for nanofibers to degrade. In light of its extended degradability and reduced water absorption, this mixed nanofiber can serve as a good skin substitute for use in skin tissue engineering [60].

#### 3.3.4 Biodegradation

**Fig. 5:**
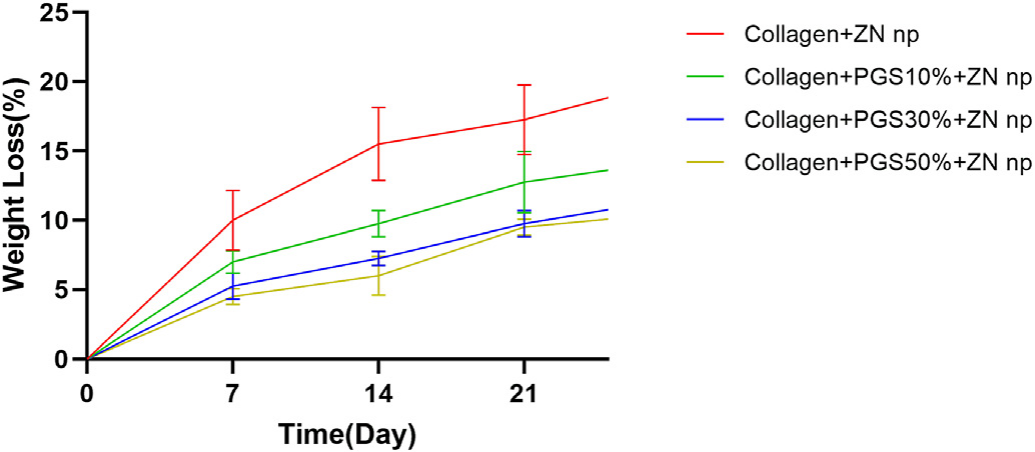
Nanofibers characterization: A, Degradation curves following soaking in PBS for Col/ZnONPs, Collagen/PGSp10%/ZnONPs, Collagen/PGSp30%/ZnONPs, and Collagen/PGSp50%/ZnONPs.

Fig. 5A shows the Collagen/PGSp scaffolds' degrading behavior. Collagen/PGSp electrospun scaffolds demonstrated a modest weight loss % after 21 days, supporting the PGSp's slow-degradation tendency as stated. Collagen nanofibers, in contrast, degraded more quickly, losing 17 1.5% of their weight by the conclusion of the trial. The proportion of degradability has reduced while the percentage of PGSp has increased in the current composition. This is consistent with the swelling information from the earlier samples. The findings demonstrated that samples with PGSp degrade at a slower pace than samples lacking PGSp. The nanofiberous structure's hydrophobic PGSp prevented water from penetrating it, hastening the structure's disintegration. Our findings are in line with those of Silva and colleagues, who mixed PGS with PCL and slowed scaffold deterioration [61].

#### 3.3.5 Tensile properties

Using universal testing equipment, the tensile characteristics of Collagen/PGSp nanofibrous scaffolds are determined (KSM-bx5450ST, Osaka, Japan). Table 2 displays the samples' stress-strain data. When exposed to dry conditions, the electrospun Collagen/PGSp 30%/ZnONPs scaffold exhibits greater force at break and elasticity while the other scaffolds exhibit much lower ones. The electrospun Collagen/ZnONPs scaffolds had a 65.213 strain at break. While the scaffolds may achieve 45 ± 2% elongation at break with a mass ratio of 70:30 for Collagen and PGSp. According to the Stress/Strain statistics for scaffolds, the scaffold with a mass ratio of 70:30 for Collagen and PGSp has higher stress/strain (0.25 mm/mm). Therefore, it appears that the scaffold structure is strengthened by the presence of PGSp in the scaffolds. The electrospun Collagen/PGSp 30%/ZnONPs fibrous scaffold exhibits a high tensile strength based on the aforementioned data in accordance with our findings, adding carbon nanotubes or tricalcium phosphate (TCP) to PGSp will undoubtedly boost its compression strength and tensile strength [62,63]. The composite tensile strength of PGSp might be greatly increased by adding silicate-based bioactive glass fibers or halloysite nanotubes [64,65].

**Table 2:** Mechanical properties of all nanofibers including Ultimate Tensile Stress(MPa), Strain (%) and Stress/Strain.

Sample	Ultimate Tensile Stress(MPa)	Strain (%)	Stress/Strain (mm/mm)
**Collagen + ZN np**	1.33 ± 0.3	65 ± 3	0.15
**Col + PGS10%+ZN np**	2.25 ± 0.2	55 ± 4	0/18
**Col + PGS30%+ZN np**	3.34 ± 0.3	45 ± 2	0/25
**Col + PGS50%+ZN np**	2.68 ± 0.5	35 ± 5	0/45

#### 3.3.6 Water contact angle analysis

**Fig. 6:**
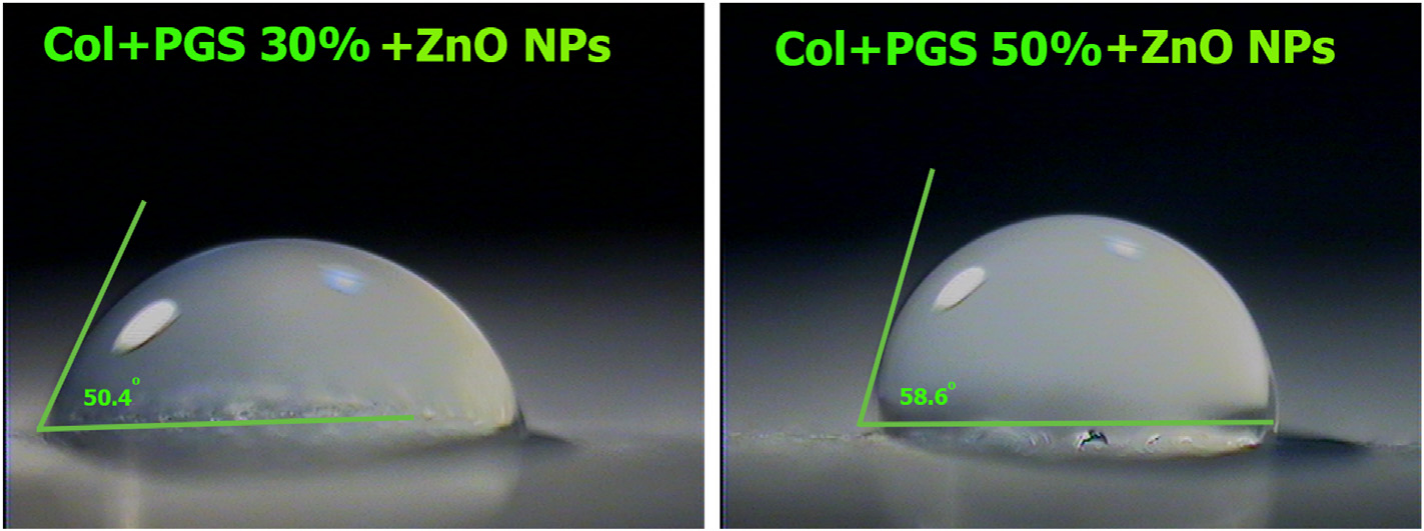
Water contact angle images of Col/ZnONPs Collagen/PGSp10%/ZnONPs, Collagen/PGSp30%/ZnONPs and Collagen/PGSp50%/ZnONPs nanofibers.

The results of the water contact angle analysis were shown in Fig. 6 to assess the wetting behavior of scaffolds with various PGSp ratios and their impact on the surface hydrophobicity of nanofibers. As shown in this picture, the ratio of PGSp decreased the ability of Collagen/PGSp scaffolds to absorb water. For the nanofibers with the corresponding Collagen/PGSp ratios of 100:0, 90:10, 70:30, and 50:50, the water contact angles were 46.2°, 48.5°, 50.4°, and 58.6°. Because PGSp is hydrophobic, as has been observed, the water-absorbing capacity of nanofibers increased as the amount of PGSp in them increased. Additionally, the chemical composition and surface shape can affect how hydrophobic the surface seems to be [48]. According to Zhang et al. dense PGSp demonstrated good hydrophilicity because hydroxyl groups were connected to the polymer backbones, and as a result, water absorbency decreased [26].

#### 3.3.7 Cell viability

**Fig. 7:**
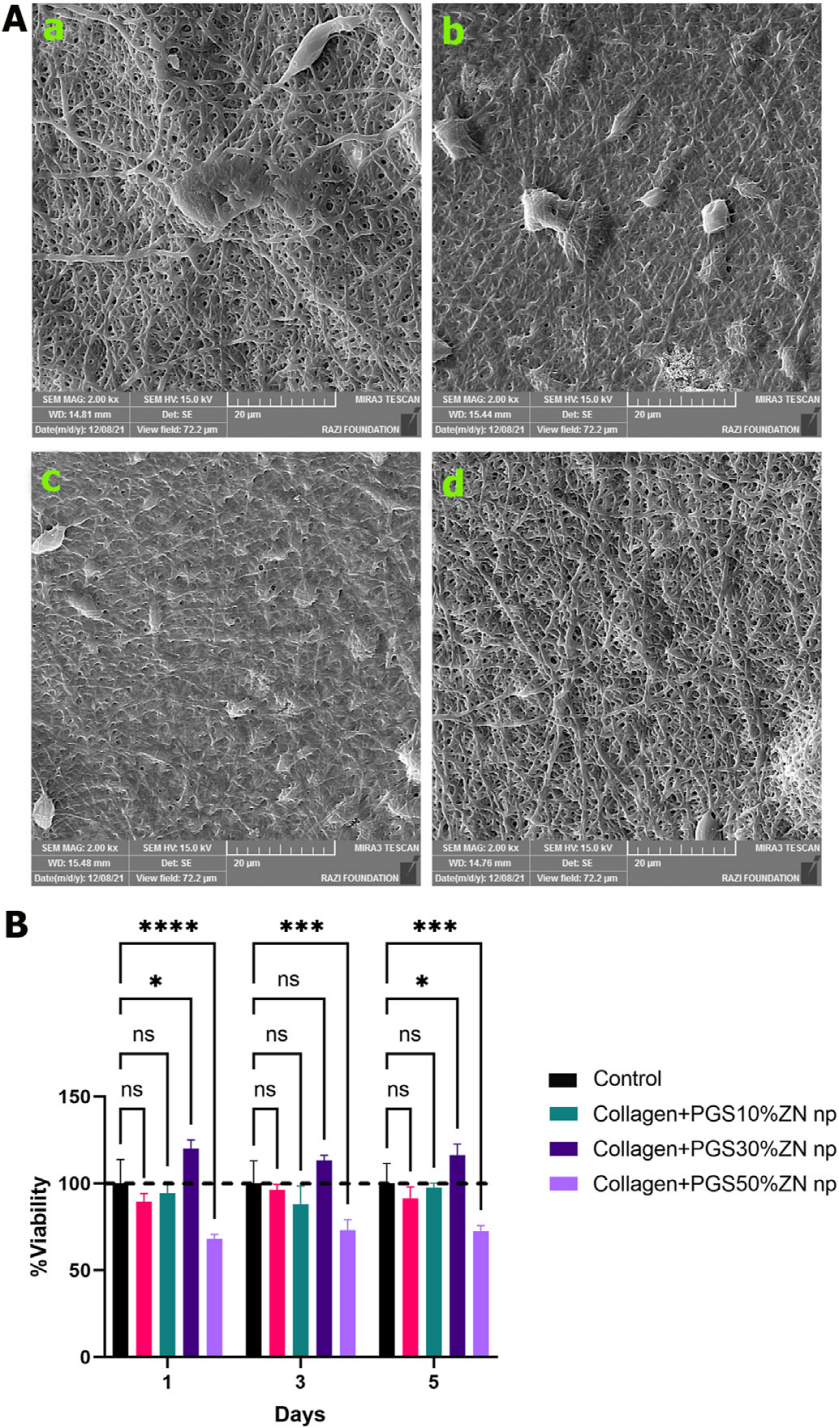
A. SEM images of Adipocyte-Derived Stem Cells (ADSC) on (a) Col/ZnONPs (b) Collagen/PGSp10%/ZnONPs, (c) Collagen/PGSp30%/ZnONPs and (d) Collagen/PGSp50%/ZnONPs nanofiber sheets. B. Results and statistical analysis of the cell viability from MTT assay for different Collagen/PGSp nanofibers containing ZnONPs (∗P-value<0.05, ∗∗ P-value<0.01, ∗∗∗ P- value < 0.001).

On the nanofibers scaffolds with various ratios of Collagen/PGSp (100:0, 90:10, 70:30, and 50:50), the MTT assay was carried out three times at intervals of 1, 3, and 5 days. In Fig. 7B, the test's findings are shown. The obtained results demonstrate that the electrospun scaffolds are fully biocompatible and that the presence of PGSp in various concentrations enhances cell adhesion and proliferation. Fig. 7B shows that other than Collagen/PGSp50%+ ZnONPs, none of the scaffolds were hazardous and that cell proliferation increased from day 1 to day 5 in all cases. On days 1 and 5, cells in the Collagen/PGSp30%+ZnONPs group grew noticeably faster than those in the control group according to Fig. 3's findings, this group had better diameter, density, and porosity than the other groups, suggesting that the scaffold has a better affinity for supporting cells.

The decrease in the scaffold's porosity may have contributed to the toxicity of Collagen/PGSp50%+ ZnONPs by increasing the mortality of the cells by decreasing their capacity to penetrate the scaffold. Similar to our findings, Saudi et al. showed that the PVA scaffolds and crosslinked PVA-PGS (40:60 and 50:50) lead to more cell proliferation than the control samples [56].

#### 3.3.8 Cell adhesion and proliferation

After 24 h, the SEM test was utilized to examine the cell adhesion and evaluate the phenotype of the cells. According to Fig. 7 cell density was almost higher in scaffolds with a higher PGSp ratio of up to 30%. In addition, cells were more numerous and adhered to the surface of the Collagen/PGSp30%+ ZnONPs scaffold more effectively. Fewer cells are linked to Collagen/PGSp30%+ ZnONPs electrospun nanofiber, as seen in Fig. 7. The toxicity of the scaffold, which was also evident in the matt results in this study, may be to blame for these outcomes. The high viscosity level of PGSp, which controls both cell attachment and proliferation, is supported by these findings [2,26].

#### 3.3.9 Microbial test

**Fig. 8:**
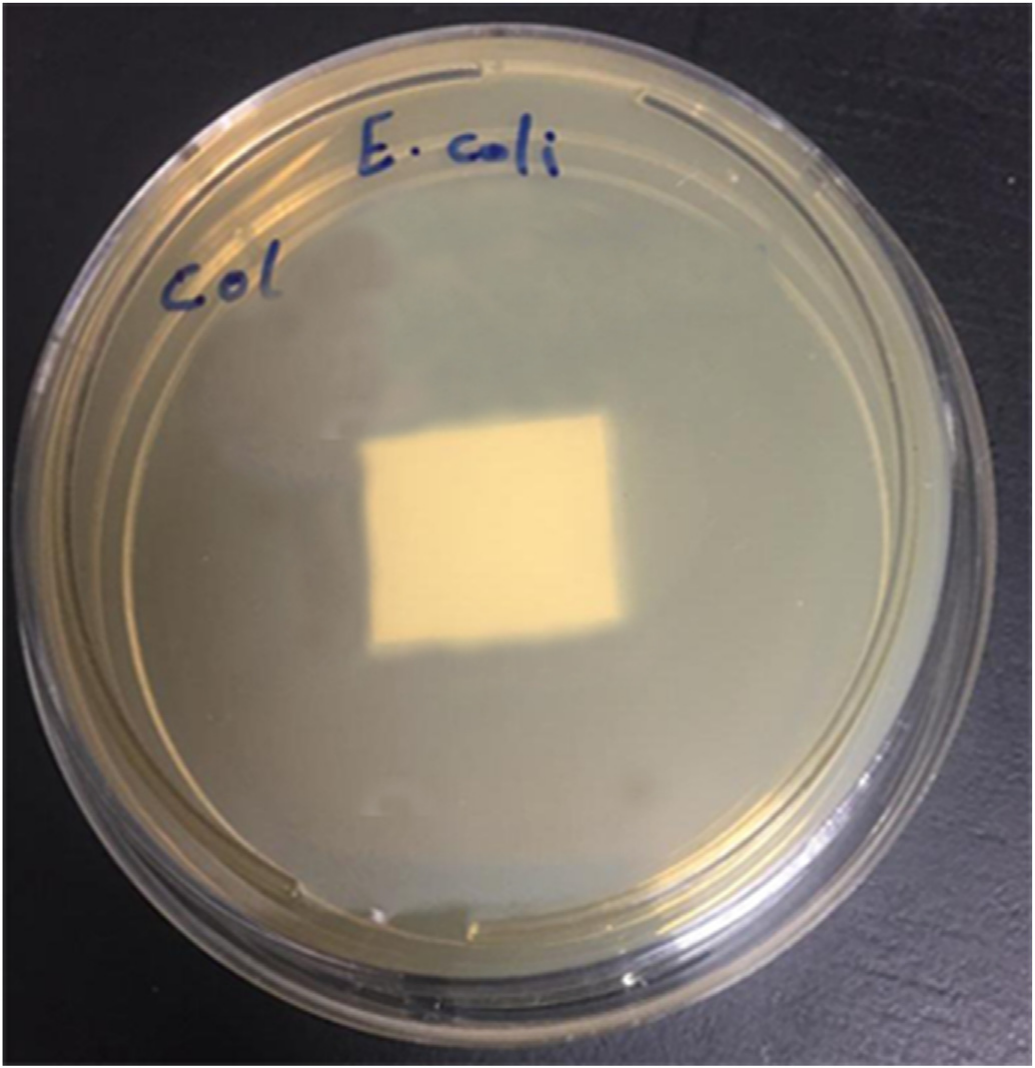
The antibacterial activity of nanofibers with contents of ZnONPs(0.1%) against A.*Staphylococcus aureus* and *E. coli* (n = 3).

In this investigation, *E. coli* and *S. aureus*, two representative Gram-positive and Gram-negative species were used to measure the growth and inhibition of antibacterial activity. Fig. 8 also depicts the in vitro antibacterial activity of the nanofiber material against *Staphylococcus aureus* and *Escherichia coli*. The nanofibers of ZnONPs were found effective at 8 mm against *Staphylococcus aureus* only and were observed at 5 mm against *Escherichia coli*. Early research indicates that ZnO nanofiber materials can effectively kill bacteria like *Escherichia coli* and *Staphylococcus aureus* by destroying their cell walls and interfering with their genetic background [66,67].

### 3.4 In vivo

Regarding to the obtained findings about the wound healing demonstrated that based on the Two-way repeated measures ANOVA statistical test, the time effect (days 0, 7, and 14) was significant (P < 0.001), meaning that, regardless to the drug treatment in different groups, with increasing days, the average wound healing percentage in rats significantly changes and evaluation time plays an important role in the increase of healing ratio percentage.

**Fig. 9:**
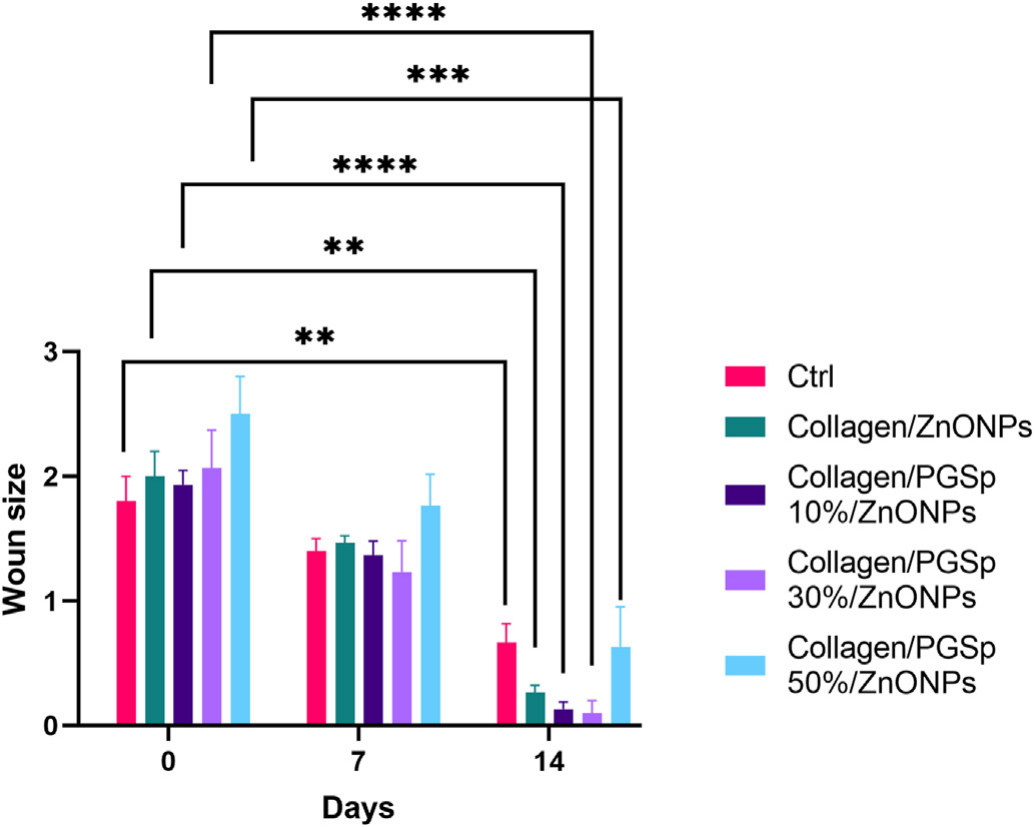
The wound sizes of in Col/ZnONPs Collagen/PGSp10%/ZnONPs, Collagen/PGSp30%/ZnONPs and Collagen/PGSp50%/ZnONPs group in days 7 and 14 in diabetic rats.

**Fig. 10:**
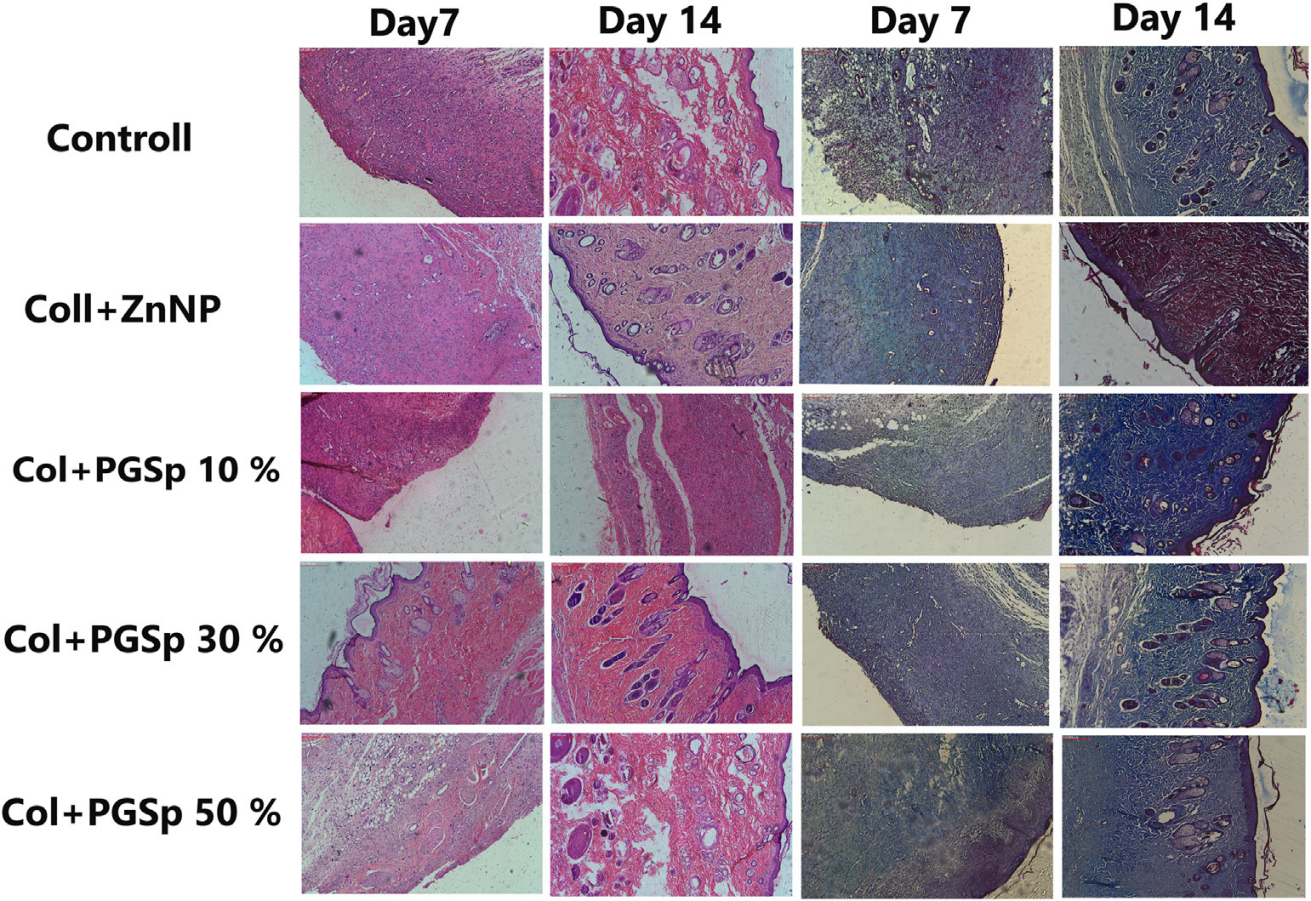
The histopathological staining concluding H&E and three chrome in days 7 and 14 in diabetic rats.

Additionally, the results of Bonferroni post hoc test also showed that regardless of the drug treatment in different groups, the average of healing in the wound surface in rats that were compared in both sides was significantly different in all three evaluation time (day 0, 7, 14) (P < 0.0001). As shown in Fig. 8 regardless of the evaluation time, the PGSp in different treatment groups had also important effect, meaning that, regardless of evaluating the time, the average wound healing percentage of rats in groups with 10% and 30% PGSp was significantly increased (P < 0.0001). Based on pathology results are shown in Table 3, Figs. 9 and 10 the severity of the parameters such as inflammatory cells, angiogenesis, re-epithelialization: migration of keratinocytes, bridging of cells and keratinization was increased in Collagen/PGSp 30%/ZnONPs group more than others at day 7. Similarly, the pathological scores were more at day 14 meaning that Collagen/PGSp 30%/ZnONPs could improve pathological condition in the treated rats. Moreover, histological evaluation by H&E staining and Masson staining showed that after applying PGS 30% at day 7 and 14, tissue structure could improve in the treated rats compared to other groups. The advantages of nanofibers as a wound dressing include enhanced hemostasis, better absorption, maintaining a wet environment by absorbing exudate, semi-permeable to stop bacterial invasion and more compatibility for wound dressing. Limitations of electrospun scaffolds contain restricted cell penetration, partial nutrient diffusion, and inadequate thickness. In this study, we tried to overcome these shortcomings with an optimal percentage of collagen and PGSp composition.

**Table 3:** The severity of the histopathological parameters such as inflammatory cells, angiogenesis, re-epithelialization: migration of keratinocytes, bridging of cells and keratinization at 7, 14 days.

Pathological scores based on the severity of the parameters at Day 7					
	CTRL	C/ZN	C/ZN/PGS 10%	C/ZN/PGS 30%	C/ZN/PGS 50%
Inflammatory cells	2	2	1.6	1.6	2
Fibroblastic cells	2	2	2.3	2.6	2.3
Collagen	1.6	1.8	2.5	2.3	2
Collagen Structure	1.3	1.6	2.3	2.6	2
Angiogenesis	2	2	2.3	2.6	1.8
Re-epithelialization: migration of keratinocytes, bridging of cells and keratinization	0	0	0	0.5	0
**Pathological scores based on the severity of the parameters at Day 14**					
	CTRL	C/ZN	C/ZN/PGS 10%	C/ZN/PGS 30%	C/ZN/PGS 50%
Inflammatory cells	2.6	2.3	1.5	1.5	3
Fibroblastic cells	2.6	2.6	3	3.5	2
Collagen	2.6	2.8	3.3	3.6	2.2
Collagen Structure	2	2.3	3	3.3	1.8
Angiogenesis	3	3.2	3	3.6	1.8
Re-epithelialization: migration of keratinocytes, bridging of cells and keratinization	3	3.5	3.6	4	2.8

## 4 Conclusion

In the current study, the blending electrospinning approach was successfully used to make Collagen and ZnONPs nanofiber with varying levels of PGSp. The objective of the research was to develop Collagen nanofiber that was properly cellular compatible and had superior mechanical, biological propertie and wound healing based on in vitro and in vivo evaluations. These results indicated the homogeneous and smooth shape and great porosity in Collagen/PGSp30%+ZnONPs scaffold. Water contact angle results showed higher hydrophobicity for scaffolds with 30%PGSp. The scaffolds with 30%PGSp nanofibers are confirmed to be biocompatible and non-toxic by the MTT assay and cell morphology. Additionally, Collagen/PGSp30%+ZnONPs nanofiber outperformed other nanofibrous scaffolds in terms of mechanical characteristics, cellular function and wound healing. For use in skin tissue engineering applications, Collagen/PGSp30%+ZnONPs nanofiber can be viewed as a potentially biocompatible scaffold.

## Funding

This research work was funded by The 10.13039/501100002660Islamic Azad University of Tehran in Iran (IR.IAU.SRB.ERC.1400.168).

## Data availability

The raw/processed data required to reproduce these findings cannot be shared at this time as the data also forms part of an ongoing study.

## Declaration of competing interest

No conflict of interest exits in the submission of this manuscript, and manuscript is approved by all authors for publication.
